# A New Role for Carbonic Anhydrase 2 in the Response of Fish to Copper and Osmotic Stress: Implications for Multi-Stressor Studies

**DOI:** 10.1371/journal.pone.0107707

**Published:** 2014-10-01

**Authors:** Anna de Polo, Luigi Margiotta-Casaluci, Anne E. Lockyer, Mark D. Scrimshaw

**Affiliations:** 1 Institute for the Environment, Brunel University, London, United Kingdom; 2 AstraZeneca, Global Health, Safety and Environment, Freshwater Quarry, Brixham, United Kingdom; CINVESTAV-IPN, Mexico

## Abstract

The majority of ecotoxicological studies are performed under stable and optimal conditions, whereas in reality the complexity of the natural environment faces organisms with multiple stressors of different type and origin, which can activate pathways of response often difficult to interpret. In particular, aquatic organisms living in estuarine zones already impacted by metal contamination can be exposed to more severe salinity variations under a forecasted scenario of global change. In this context, the present study aimed to investigate the effect of copper exposure on the response of fish to osmotic stress by mimicking in laboratory conditions the salinity changes occurring in natural estuaries. We hypothesized that copper-exposed individuals are more sensitive to osmotic stresses, as copper affects their osmoregulatory system by acting on a number of osmotic effector proteins, among which the isoform two of the enzyme carbonic anhydrase (CA2) was identified as a novel factor linking the physiological responses to both copper and osmotic stress. To test this hypothesis, two *in* *vivo* studies were performed using the euryhaline fish sheepshead minnow (*Cyprinodon variegatus*) as test species and applying different rates of salinity transition as a controlled way of dosing osmotic stress. Measured endpoints included plasma ions concentrations and gene expression of CA2 and the α1a-subunit of the enzyme Na^+^/K^+^ ATPase. Results showed that plasma ions concentrations changed after the salinity transition, but notably the magnitude of change was greater in the copper-exposed groups, suggesting a sensitizing effect of copper on the responses to osmotic stress. Gene expression results demonstrated that CA2 is affected by copper at the transcriptional level and that this enzyme might play a role in the observed combined effects of copper and osmotic stress on ion homeostasis.

## Introduction

Ecotoxicological studies on environmental chemicals are usually performed under optimal exposure conditions, whereas aquatic organisms in their natural settings have to cope with additional stressors, such as variations in temperature, oxygen levels or salinity, which can affect the way they respond to chemical stressors [1]. Conversely, exposure to chemical stressors could impair organisms’ responses to changes in environmental factors. In either cases, this two-way interaction may or may not lead to more adverse effects on the organisms and ultimately on the population, since the correlation between tolerance to chemical and non-chemical stressors can be positive or negative, depending on the type and level of stressors in question (e.g. [2], [3]).

The potential interactions between toxic substances and environmental factors represent one of the main challenges for ecotoxicologists. The importance of refining toxicity studies and applying them to more complex scenarios has been recognized by both the EU and US EPA, who have expressed an increasing interest in studying the responses of biological systems to a combination of stressors, both chemical and environmental, rather than to single chemical in stable conditions [4], [5]. An integrated examination of chemical and non-chemical stressors is especially pertinent when considering chemical pollution in the context of global change scenarios, where more fluctuating environmental variables are plausible to influence the responses of biological systems to chemical exposure [6], [7]. This is again a two-way interaction, because global change can make organisms more sensitive to chemical stressors as well as exposure to chemical stressors can make organisms more sensitive to changes in environmental stressors caused by global change.

In this context, the challenge is to identify physiology-based interactions between non-chemical and chemical stressors affecting key physiological processes in an organism. As a first step to applying a multi-stressor approach into ecotoxicological studies, it is crucial to acquire an understanding of the mechanisms of action of the stressors in question, by dissecting the biological pathways through which they exert their effects [8]. One possible approach in this sense is the application of the adverse outcome pathway (AOP) concept, a unified framework that links molecular initiating events with the cascade of responses occurring across all levels of biological organization [9]. Such approach, despite being mainly qualitative [10], does highlight the importance of dissecting the physiological mechanisms and toxicodynamic processes underpinning the complexity of many biological responses, which is a key step in interpreting the stress-response dynamics displayed by any biological system in the complexity of the real world.

With this aim in mind, the present study examined the mechanisms of interaction between one chemical stressor, i.e. copper, and one environmental stressor, i.e. osmotic stress, which was applied in the form of salinity transitions from either freshwater to saltwater or from saltwater to freshwater. In order to put this laboratory-based study into an environmentally realistic context, we chose to investigate copper-salinity interactions by mimicking under laboratories conditions the salinity changes occurring in estuaries, which are environments where the combination of anthropogenic impacts and fluctuating abiotic factors represents an ideal context to study the interactions between chemical and non-chemical stressors. Considering future global change scenarios and their impacts on estuarine and coastal pollution [11], aquatic organisms inhabiting transitional environments are likely to be exposed to more severe and/or more frequent salinity fluctuations in the future, as a result of increased frequency of extreme events [12]. Therefore salinity changes, also defined as osmotic stress, were chosen as the non-chemical stressor to test in this study. As for the chemical stressor, we selected a metal (i.e. copper) because in general metal contamination is of particular concern in estuarine and coastal environments, where the historically high anthropogenic impacts, mainly due to shipping, urbanization and industrialization, often lead to elevated concentrations of metals both in the water column and in the sediments [13]. In particular, one of the main concerns about transitional zones is represented by copper, whose use as biocide in antifouling painting coatings has increased since TBT (tributyltin) and other organic biocides has been phased out [14]. We therefore focused on the interactions between copper and salinity changes, given their theoretical as well as applicative relevance for multi-stressor studies applied to transitional environments in a global change perspective. However, since this topic has been almost exclusively addressed from the point of view of the effects of different salinities on copper toxicity [15]–[18], we instead put more emphasis on the effects of copper exposure on the response of aquatic organisms, i.e. fish, to salinity transitions. In accordance with the mechanistic intent of the study, we hypothesized that, given that acute copper toxicity is mainly a consequence of osmotic disruption [19]–[21], copper-exposed fish are more sensitive to osmotic stresses, as copper can affect their osmoregulatory system by interacting with a number of osmotic effector proteins, such as the enzyme Na^+^/K^+^ ATPase (e.g. [22]). As we previously discussed in a review on copper toxicity in saline environments [23], a critical screening of the ecotoxicological data available in the literature on copper toxicity and salinity led to the hypothesis that one factor linking the main physiological responses to both copper and osmotic stress is the cytosolic isoform-2 of the enzyme carbonic anhydrase (CA2), potentially a copper target [24]–[26] with salinity dependent expression and activity [27]–[29]. Because CA2 displays its osmoregulatory functions not only in the gills, but also in the intestine of fish [30], we argued that in saline environments the intestine should be considered alongside the gills as a site of action for copper [23]. We hypothesized that the combined effect of copper and osmotic stress is not the overall product of two independently acting factors, but rather the outcome of a mechanistic interaction between two stressors that act through similar pathways and affect similar effector proteins.

The *in* *vivo* experiments reported here were aimed at testing that hypothesis, using the euryhaline fish sheepshead minnow (*Cyprinodon variegatus*) as test species and applying different types and rates of salinity transitions as a way to administrate different doses of osmotic stress. Results of plasma sodium levels measured before and after the salinity transitions overall supported the hypothesis of a copper-disrupted response to osmotic stress, whilst gene expression results provided a mechanistic understanding of the copper-salinity interaction and supported the argument that the CA2 enzyme plays a role in the combined responses to copper and osmotic stress.

## Materials and Methods

### Ethics statement

These studies were carried out under project and personnel licences granted by the Home Office under the United Kingdom Animals Act (Scientific Procedures).

### Experiment 1

#### Test species

Adult male and female sheepshead minnows (*Cyprinodon variegatus*) were obtained from the Brixham Environmental Laboratory (Brixham, UK) and acclimated to freshwater for approximately three weeks under flow-through conditions. Fish age was 4 month post-hatching and average wet weight was 4.7±1.1 g.

#### Experimental design and protocol

The experiment was carried out using a continuous flow-through system. Thermostatically heated dechlorinated carbon filtered tap water, from a header tank, flowed through 6 flow-meters into 6 mixing chambers via silicon tubing (medical grade, VWR) at a rate of 120 ml/min. From each mixing chamber the water flowed into the fish tanks through silicone tubing. In total there were 6 glass fish tanks each with a working volume of 11 L. Each tank was aerated and received approximately 15 tank volume renewals per day. During the exposure period, copper was added as CuSO_4_·5H_2_O dissolved in 1% HNO_3_ (Optima grade, Fisher Scientific) and dosed into each individual mixing chamber via a peristaltic pump at a rate of 0.12 ml/min. The stock solutions and mixing regime were designed to yield final nominal copper concentrations of 10 and 100 µg/L. Dilution water and chemical flow rates were checked twice per day and adjusted if necessary. Temperature, pH, oxygen, total alkalinity (see Table S1), nitrate and ammonia were monitored and recorded daily. Water samples for total organic carbon (TOC), copper and major constituents analyses were collected in 50 ml centrifuge tubes on days 3, 6, 8 and 9. Samples for TOC analysis were stored at −20°C after collection, whereas samples for copper and major constituents analysis were acidified with 1% HNO_3_ and stored at 4°C.

The 9-day exposure experiment consisted of 3 treatments: 0, 10 µg/L and 100 µg/L nominal copper concentrations. All treatments were in freshwater (FW) for the first 8 days of exposure. Each treatment was composed of 2 single-sex tanks, each tank containing 6 fish (12 fish per treatment, 6 males and 6 females). Fish were maintained in a photoperiod of 16 h of light followed by 8 h of dark, with 20 min dawn/dusk transition, and were fed *ad libitum* three times per day: twice with flake food (King British Tropical flake food, Lillico, Surrey) and once with brine shrimp (Tropical Marine Centre, gamma irradiated). Food was withheld 20 h prior to each sampling. After 8 days of exposure in FW, 3 fish per tank (6 fish per treatment) were humanely sacrificed according to UK Home Office procedures, using an overdose of buffered ethyl 3-aminobenzoate methanesulfonate (300 mg MS222/L, adjusted to pH 7.8, the average pH throughout the study). Blood samples were collected from the caudal peduncle using heparinised capillary tubes, transferred into Eppendorf tubes and kept on ice until plasma was separated by centrifugation at 14,000 *g* for 5 min, removed and stored at −20°C. Fish were weighed and measured (fork length). Tissue samples (gills, liver and intestine) were collected by dissection. The intestine was divided into three segments (anterior, mid and posterior intestine) and sub-samples of each segment were kept for gene expression analysis. This procedure was chosen in order to account for potential differences in gene expression along the intestinal tract. All tissue samples were immediately frozen in liquid nitrogen after collection and stored at −80°C.

#### Salinity transition

After the first sampling on day 8, the salinity in all treatments was increased from 0 to 20 ppt over a time of 4 h by dosing hyper-concentrated (200 ppt) saltwater (SW) prepared by dissolving and mixing synthetic seasalts (Tropic Marin) in three glass tanks of 40 L volume each, filled with dechlorinated carbon filtered tap water. The brand of synthetic seasalts (Tropic Marin) used to prepare the saline stock solutions was selected as it has been shown to have a low content of trace elements, particularly copper, compared to other commercially available brands [31]. The hyper-saline stock solution was prepared 24 h in advance and left overnight with strong aeration to allow the pH to stabilize. SW was dosed from the stock solution into the mixing chambers at a rate of 12 ml/min to yield a final salinity of 20 ppt in all fish groups. Salinity was monitored every 15 min with a refractometer throughout the salinity transition period. After reaching 20 ppt, fish in all treatments were held in SW for 24 h, until the second sampling on day 9. Copper dosing was maintained constant during the entire study. On the 9^th^ day of exposure, the remaining fish (6 per treatment) were sampled following the same procedure described above, with the exception of the MS222 solution, which was prepared in SW instead of FW and buffered at a pH of 8.4 (instead of 7.8), consistent with the salinity and average pH of the tanks after the salinity transition.

### Experiment 2

#### Test species

Adult male and female sheepshead minnows (*Cyprinodon variegatus*) were obtained from a livestock bred and maintained at Brunel University (UK) in a SW re-circulated system supplied with UV-sterilizer, sand biofilter and protein skimmer (Marine Compact Filtration System, Tropical Marine centre). The synthetic seasalts (Tropic Marin) used for maintaining the system were the same used for the preparation of the artificial SW during the two experiments.

Approximately one month before the start of the experiment, a sub-stock of fish has been gradually moved to FW and then kept under these conditions for three weeks before starting the exposure. Fish age at the beginning of the experiment was approximately 6 months post-hatching and average wet weight was 2.7±0.7 g.

#### Experimental design and protocol

The experiment was carried out using a continuous flow-through system and artificial SW dosed from a concentrated stock for the groups held in SW. Thermostatically heated dechlorinated carbon filtered tap water, from a header tank, flowed through 16 flow-meters into 16 mixing chambers via silicon tubing (medical grade, VWR) at a rate of 60 ml/min. This final flow of 60 ml/min was half the rate of the one used in the Experiment 1 (120 ml/min). Thus, a full replacement was now reached within double the time, which implied that the salinity switch on day 20 would then be performed over 8 h instead of 4, hence representing a milder osmotic stress, but still within the time range of a tidal change in an average estuary. From each mixing chamber the water flowed into the fish tanks through silicone tubing. In total there were 16 glass fish tanks with a working volume of 11 L. Each tank was aerated and received approximately 7 tank volume renewals per day. During the exposure period, copper was added as CuSO_4_·5H_2_O dissolved in 1% HNO_3_ (Optima grade, Fisher Scientific) and dosed into each individual mixing chamber via a peristaltic pump at a rate of 0.06 ml/min. The copper stock solutions and mixing regime were designed to yield final nominal copper concentrations of 32, 100 and 320 µg/L. Dilution water and chemical flow rates were checked twice per day and adjusted if necessary. Temperature, pH, oxygen, total alkalinity (see Table S2), nitrate and ammonia were measured and recorded daily. Water samples for TOC, copper and major constituents analysis were collected in 50 ml centrifuge tubes on days 1, 4, 8, 12, 16, 19 and 21. Water samples for TOC analysis were stored at −20°C after collection and water samples for copper and major constituents analysis were acidified with 1% HNO_3_ and stored at 4°C.

The 21-day exposure experiment consisted of 4 treatments: 0, 32, 100 and 320 µg/L nominal copper concentrations (refer to Figure S2 for an outline of the experimental design and set-up). Each treatment consisted of 4 tanks, 2 in FW (0 ppt) and 2 in SW (20 ppt), of which one replicate tank contained 10 males and one 10 females (*n = *12 in control tanks). SW conditions were maintained by dosing hyper-concentrated SW (100 ppt) from a dosing stock prepared daily by dissolving and mixing synthetic seasalts (Tropic Marin) in two fibreglass tanks of 80 L volume each, filled with dechlorinated carbon filtered tap water. The hyper-saline stock solution was made at least 24 h in advance and left overnight with strong aeration to allow the pH to stabilize. SW was transferred into two intermediate dosing stocks (40 L volume each) and from there dosed into the mixing chambers at a rate of 12 ml/min to yield a final salinity of 20 ppt in the SW groups (2 tanks per treatment). Throughout the experiment, fish were maintained under the same photoperiod and food regime as in the Experiment 1.

After 19 days of copper exposure in either FW or SW, half of the fish (10 per treatment, 12 from controls) were humanely sacrificed according to UK Home Office procedures, using an overdose of ethyl 3-aminobenzoate methanesulfonate (300 mg MS222/L, buffered at pH 7.6, the average pH throughout the study, and adjusted to a salinity of either 0 or 20 ppt, consistently with the exposure conditions). Blood samples were collected and stored as indicated for Experiment 1. Fish were weighed and measured (fork length). Tissue samples of gills and intestine were collected by dissection. The intestine was divided into two segments (mid-anterior and posterior) and only sub-samples of the mid-anterior segment were kept for gene expression analysis, since preliminary gene expression analyses had shown that the mid-anterior segment displayed slightly higher expression levels of the measured genes, compared to the posterior segment of the intestinal tract (data not shown). All tissue samples were immediately frozen in liquid nitrogen after collection.

#### Salinity transition

On day 20 of exposure (after the first sampling), the salinity in all treatments was either increased from 0 to 20 ppt in the groups previously held in FW or decreased from 20 to 0 ppt in the SW groups, over a time of 8 h. This was achieved by swapping the final tubing between the mixing chambers and the fish tanks (Figure S2), with a time lag of two hours between treatments, in order to assure that each group would be held in the new conditions for the same time (24 h), assuming a sampling rate of 10 fish per hour on the following day. Salinity was monitored at least every hour with a refractometer throughout all the salinity switch period (see Figure S1). After reaching either 0 or 20 ppt in all groups, fish were held in the new conditions for 24 h until the second sampling. Copper dosing was maintained constant during the entire study. On day 21 of exposure, the remaining fish (8 to 10 per treatment, 12 from controls) were sampled following the same procedure described for Experiment 1.

### Chemical analyses and speciation modelling

#### Copper, total organic matter and major cations in water

Total copper concentrations in fish tank water were determined by either graphite furnace, GF-AAS (4100ZL Zeeman Atomic Absorption Spectrometer, Perkin Elmer) or flame atomic absorption spectroscopy, F-AAS (AAnalyst100 AAS, Perkin Elmer), depending on the expected range of concentrations, using standard operating conditions and dilutions when necessary. For analytical procedures, all acid was nitric acid (Optima grade, Fisher Scientific). In order to account for sodium interference in the SW series, calibration standards were prepared in acidified Milli-Q water, with the addition of artificial seasalts to yield a final salinity of 20 ppt (or lower, when samples were diluted). Copper standards were run every 6 samples to check measurement accuracy. Total organic carbon (TOC) was determined as the Non-Purgeable Organic Carbon fraction (NPOC) by high-temperature catalytic oxidation using a Shimadzu total organic carbon-V CPN Analyzer. Major cations (Na^+^, Mg^2+^ and Ca^2+^) concentrations in water samples were determined by F-AAS after appropriate dilution.

#### Copper speciation modelling

Based on water parameters, measured TOC, cations and total dissolved copper concentrations (Table S1, S3 and S4), inorganic and total speciation of copper in exposure conditions (both in FW and SW) was calculated using the Visual MINTEQ 3.0 software [32]. For the total speciation calculations, charges on dissolved organic matter were calculated based on speciation and the % of organic C was set at 50.

#### Copper burden in liver

Six individual 0.2 g aliquots of certified standard DOLT-4 (dogfish liver certified reference material for trace metals, NRC, Canada) were pre-digested overnight in 4 ml of 100% HNO_3_, digested in a Microwave Accelerated Reaction System (MARS X, CEM Corporation) following the heating programme recommended by the manufacturer for fish tissue digestion (15 min ramp-to-temperature time and 15 min hold time at 200°C), diluted appropriately and analysed by F-AAS. The average wet weight of the liver samples was 0.1±0.06 g. For each batch of samples, one blank and one certified standard were analysed and the average percent recovery was 119±19% (*n* = 5).

#### Copper content in fish food

Copper content in fish food was analysed by GF-AAS after pre-digestion with HNO_3_, microwave digestion (15 min ramp-to-temperature time and 15 min hold time at 200°C) and appropriate dilution with Milli-Q water.

#### Copper, chloride and major cations in plasma

Plasma copper concentrations were determined by GF-AAS after dilution in acidified Milli-Q water (1% HNO_3_). Plasma Na^+^, Mg^2+^ and Ca^2+^ concentrations were measured by F-AAS and plasma Cl**^−^** concentrations by ion chromatography (DIONEX DX, Dionex Corp.), in both cases after appropriate dilution.

### Bioinformatics and molecular analyses

#### RNA extraction and cDNA synthesis

Total ribonucleic acid (RNA) was isolated from individual gills and intestine samples (mean tissue weights were respectively 25.8±8.6 and 17.7±9.2 mg) using the RNeasy Fibrous Tissue Mini Kit (Qiagen), according to the manufacturer’s instructions, which included tissue homogenization in buffer RLT using a TissueLyser II (Qiagen) for 3 min at maximum speed. The protocol also included a DNAse step to eliminate contaminating genomic DNA. The extracted RNA was resuspended in 50 µl of RNase-free water. Quantity and purity of each RNA sample were determined by spectrophotometry (Nanodrop, Fisher Scientific), and RNA integrity was visually checked by agarose gel electrophoresis. Complementary DNA (cDNA) was synthesised from 2 µg total RNA using Invitrogen SuperScript III First-Strand Synthesis System for reverse transcription-PCR kit according to the manufacturer’s protocol, using random hexamers to prime synthesis. Diluted (1∶5) cDNA samples were assessed for carbonic anhydrase isoform-2 (CA2) and Na^+^/K^+^ ATPase α1.a5-isoform (NKA) expression using quantitative real-time PCR (qPCR).

#### NKA and CA2 primer design

Sheepshead minnow specific qPCR primers were developed for both NKA and CA2 from partial *C. variegatus* mRNA sequences identified from GenBank using basic local alignment search tool (BLAST) searches. For NKA, no previously characterized sequence of *C. variegatus* NKA was available in the National Centre for Biotechnology Information (NCBI) databases (http://www.ncbi.nlm.nih.gov/). However, the screening of NCBI Expressed Sequence Tags (ESTs) database led to the identification of four highly similar *C. variegatus* ESTs (GenBank Acc. No. GE337281.1, GE337212.1, GE334919.1 and GE336240.1). GE337281.1 demonstrated 79% identity with the 3′ region of the NKA sequence expressed in *Oncorhynchus mykiss* and in BlastX searches it identified NKAα1-isoforms from several species, including *Danio rerio* (Acc. No. AAH90285), which showed high similarity with *C. variegatus* EST and hence confirmed its identity. For CA2, a partial mRNA sequence of *C. variegatus* putative cytosolic carbonic anhydrase (cCA) (Acc. No. HM142344.1) had been previously identified. HM142344.1 showed 81% similarity with the 5′ region of the EST of CA2 expressed in *Pimephales promelas* (Acc. No. DT261041.1), which in turn identified *Oncorhynchus mykiss* carbonic anhydrase isoform-2 (NP_954685) in BlastX searches, confirming its identity and hence that of the original *C. variegatus* sequence. These identified sequences were used to design the primers for NKA and CA2 (Table 1), with the assistance of PRIMER3 web software (http://bioinfo.ut.ee/primer3-0.4.0/).

**Table 1:** Primers sequences.

Gene	Forward Primer	Reverse Primer	T (°C)
CA2	5′- GAAGGTTCTGGATGCTTTGG - 3′	5′- AGTTGGAGAAGGTGGTCTGC - 3′	59
NKA	5′- GCCACACAGCCTTCTTCAC – 3′	5′- ACAATAGAGTTCCTCCTGGTCTTG – 3′	59
18S	5′– GCTGAACGCCACTTGTCC – 3′	5′– CTCAGAGCAAGCAATAGCCTTA – 3′	57

Primers used for qPCR and respective optimal annealing temperatures.

#### NKA and CA2 gene expression

The qPCR primers designed for *C. variegatus* CA2 and NKA were verified using PCR. Reactions contained diluted gill/intestine cDNA (1∶5), 1× Buffer, 1.5 mM MgCl_2_, 0.2 mM dNTPs mix, 0.5 µM forward and reverse primer and 0.1 µM *Taq* Polymerase. Cycling conditions were 2 min initial denaturation at 95°C, 35 cycles at 95°C for 30 sec, annealing at 58/62°C for NKA/CA2 respectively, then 72°C for 20 sec and final extension of 5 min at 72°C. PCR products were electrophoresed on 2% agarose gel containing GelRed (Biotium) to verify a single product and correct amplicon size. qPCRs for CA2, NKA and the reference gene 18S were performed in triplicate on cDNA from individual fish using a CFX96 Real-Time PCR detection system (Bio-Rad) and Fast SYBR Green Master Mix (Invitrogen) as per instructions of the manufacturer. Reactions were optimized for annealing temperature (see Table 1) and run for 2 min at 50°C followed by 10 sec at 95°C, then 40 cycles of 10 sec at 95°C, 30 sec at optimal annealing temperature and then 1 min ramp from 55 to 65°C. Finally a dissociation curve was obtained (melt curve: 65 to 95°C, increment 0.5°C for 0.05 sec) to confirm single products in each reaction. The relative expression of the target genes were normalized to the expression of the housekeeping gene 18S using the Excel-based software qGENE [33], which takes into account the amplification efficiency of both the target genes and the reference gene to calculate the mean normalized expression (MNE) of each target gene.

### Statistical analysis

Numerical data are presented as means ± SD throughout. Statistical analyses were conducted using SigmaStat software (version 3.5 Systat Software Inc.). Data were analysed for normality (Kolmogorov-Smirnov test) and variance homogeneity (Levene’s test). Where assumptions of normality and homogeneity were met, one-way analysis of variance (ANOVA) was performed, followed by all pairwise comparison using Dunn’s *post-hoc* test. Where the assumptions were not met, data were analysed using a non-parametric test, Krustal-Wallis ANOVA on ranks, followed by Dunn’s *post-hoc* test. Significant differences between pre- and post-salinity transition data were assessed by the Tukey *t*-test. In all cases, values were considered significantly different at *P*<0.05.

## Results

### Experiment 1

#### Water chemistry and copper speciation modelling

Average copper concentrations in the water during the exposure period were <1 µg/L in the controls, 10.04±0.77 in the low-copper and 115.5±9.97 µg/L in the high-copper treatment (Table S4).

The average concentration of organic matter in the water was 2.6±0.5 mg/L and remained stable throughout the exposure period, ranging between 1.8 and 3.9 mg/L. Among the water parameters (Table S1), alkalinity and pH underwent the most significant increase following the salinity switch, probably as a result of the increased amount of calcium carbonate in the water due to the dissolved seasalts used to prepare the SW stock solutions [31]. Higher calcium carbonate concentrations shifted the carbonate equilibria towards more basic conditions and this change, along with the increased water concentrations of cations and chloride (Table S3), affected the inorganic speciation of copper, as it can be observed in Table 2. The most relevant difference in copper speciation between pre and post salinity switch was the decreased percentage fraction of free copper ions (from around 28% to 14% of the inorganic speciation), which are considered the most bioavailable and therefore toxic form. This fraction and the other percentage fractions of copper forms reported in Table 2 are calculated without including organic matter among the input parameters. When factoring it into the calculations, the model predicted that, given the same average amount of organic matter, around 90% and 84% of the total copper was bound to it, respectively in FW and SW (Table 3). Hence, although it decreased in SW, the organic fraction of copper remains the most represented fraction. As this form of copper is considered not bioavailable, internal copper concentrations, both in the liver and in the plasma, were measured in order to confirm that copper was indeed uptaken by the fish and its internal levels displayed a dose-response trend.

**Table 2:** Inorganic copper speciation.

Inorganic copper speciation		
Species	% of total copper	
	FW	SW
Cu^2+^	28.2	14.2
Cu(OH)^+^	55.3	44.5
Cu(OH)^2^	7.44	14.07
Cu_2_(OH)_2_^2+^	4.44	4.53
CuCl^+^	0.02	2.28
CuCO_3_	3.75	18.26

Most relevant copper forms are reported (expressed as % of total dissolved copper concentrations), repectively in FW and SW, as calculated by Visual MINTEQ using measured water chemistry parameteres of Experiment 1.

**Table 3:** Total copper speciation.

Total copper speciation		
Species	% of total copper	
	FW	SW
Cu^+2^	3.08	2.46
Cu(OH)^+^	6.02	7.71
Cu(OH)^2^	0.81	2.44
Cu_2_(OH)_2_^2+^	0.05	0.14
CuCO_3_	0.41	3.17
Organic fraction	89.6	83.6

Most relevant copper forms are reported (expressed as % of total dissolved copper concentrations), repectively in FW and SW, as calculated by Visual MINTEQ using measured water chemistry parameteres of Experiment 1.

#### Liver copper burden and copper content in food

**Figure 1:**
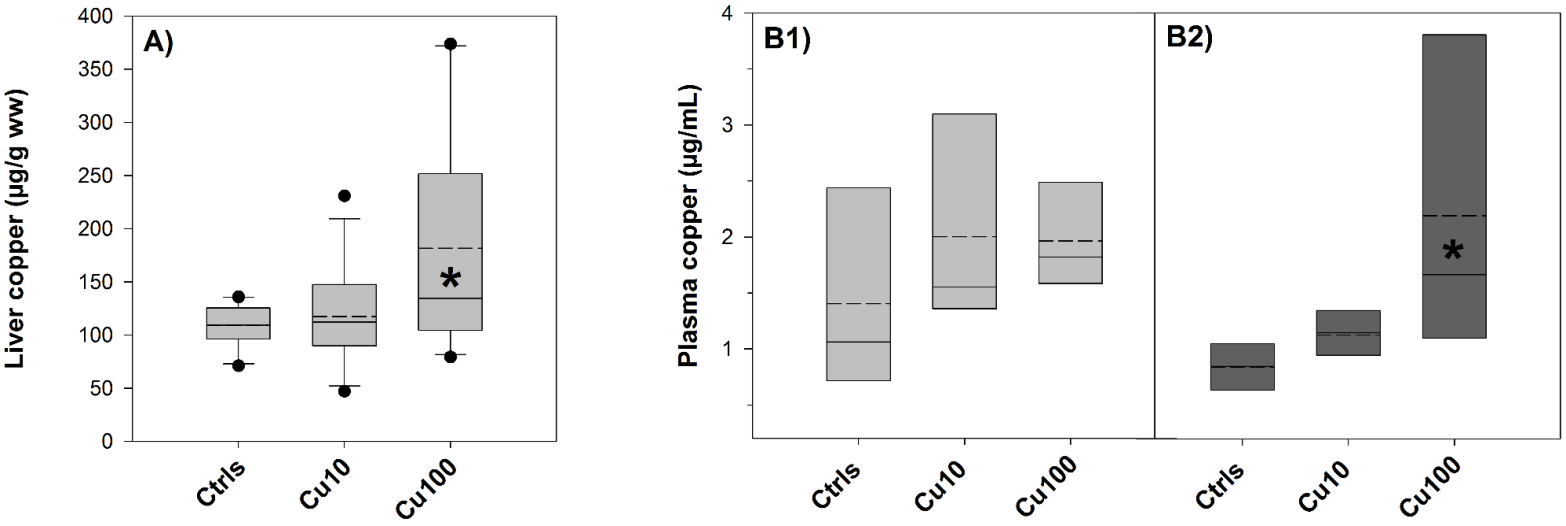
Copper in liver and plasma – Exp.1. (**A**) Copper liver burden (expressed as µg/g wet weight) in fish exposed to 0 (Ctrls), 10 and 100 µg/L copper for 9 days. Values are means ± SE (*n* = 10/12). The asterisk denotes statistically significant difference from the control value (*P*<0.05, Tukey *t*-test) (**B1–2**) Plasma copper levels (µg/ml) in fish exposed to 0, 10 and 100 µg/L copper and sampled either before (B1) or after (B2) the salinity transition. Values are means ± SE (*n* = 4/7). The asterisk denotes statistically significant difference from the control value post switch (*P*<0.05, Tukey *t*-test). Solid horizontal lines represent median values and dashed lines represent mean values.

Hepatic copper levels in the high-copper treatment were significantly elevated after 9 days of exposure (Figure 1). However, the relatively high copper levels measured in controls fish (around 110 µg/g wet weight), given the low concentrations of copper in the water (<1 µg/L), led us to investigate fish food as a potential source of copper in control fish. Analysis showed that copper concentrations were 9.1±3.2 µg/g wet weight in the flakes and 1.5±0.8 µg/g wet weight in the brine shrimps, thus providing a possible explanation for the elevated hepatic copper levels in control fish.

#### Plasma copper

Despite model predictions indicating that most of the total dissolved copper was probably complexed by organic matter, 8 days of copper exposure resulted in an increase in plasma copper levels detectable at 10 µg/L and statistically significant at 100 µg/L post salinity switch (Figure 1B). Within the same treatment, plasma copper levels did not show a significant difference pre and post switch.

#### Plasma ions

**Figure 2:**
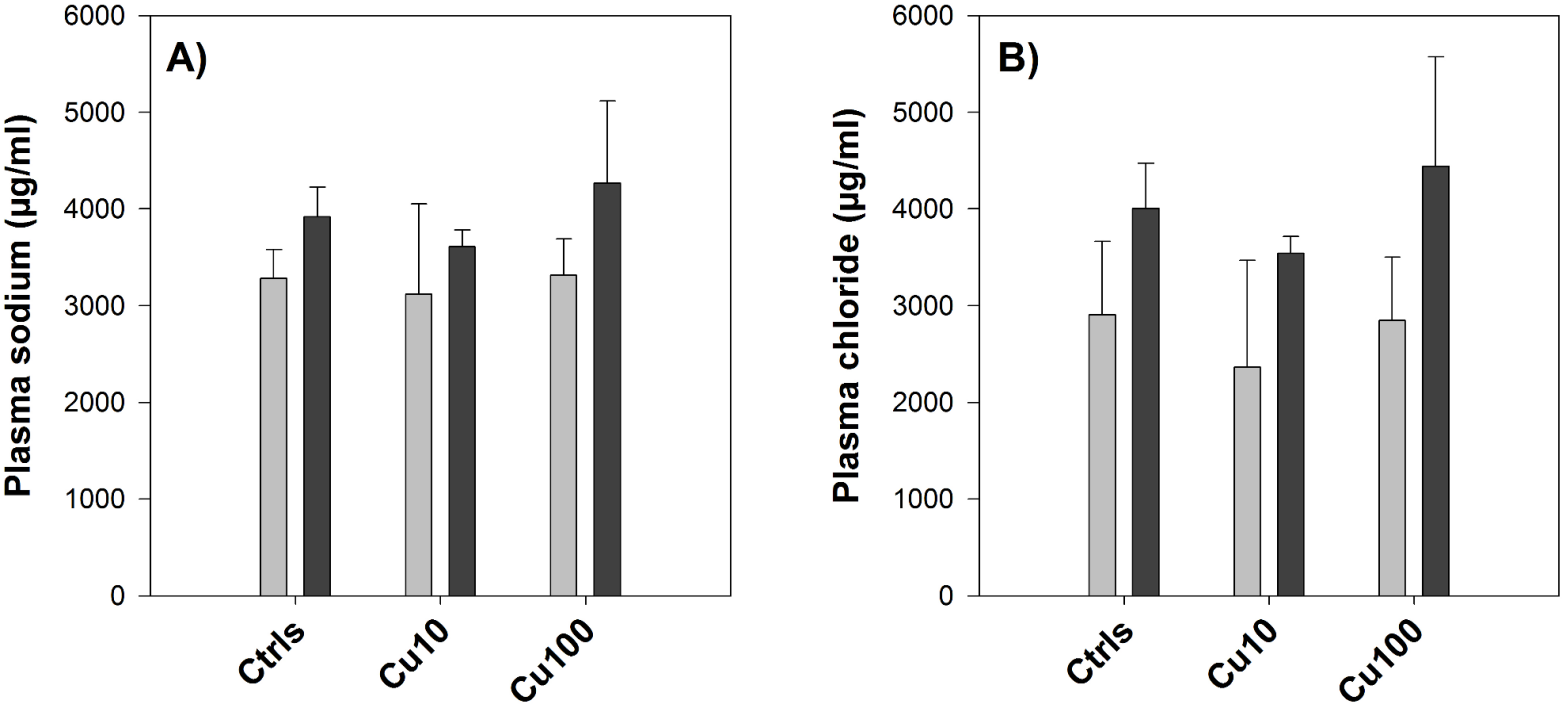
Plasma sodium and chloride – Exp.1. Plasma sodium (A) and chloride (B) levels (µg/ml) measured in fish exposed to 0 (Ctrls), 10 and 100 µg/L copper before (light grey bars) and after (dark grey bars) the salinity transition. Values are means ± SE (*n* = 6/7).

Plasma sodium levels were around 3000 µg/ml in control fish after 8 days of exposure in FW and did not appear to be significantly affected by copper exposure in a concentration-dependant manner. However, the transition to SW resulted in an average 30% increase of sodium levels in the high-copper treatment, whilst only a 20% increase was detected in the controls. Plasma chloride levels followed a similar trend, displaying a 38, 50 and 56% increase after the salinity transition respectively in controls, Cu10 and Cu100 (Table 4). Plasma magnesium levels underwent a similar though smaller change, whereas calcium cations appeared to follow an opposite trend, showing a greater degree of change in the controls compared to the copper-exposed fish. However, none of the changes in plasma ions levels were statistically significant (Figure 2).

**Table 4:** Degree of change in plasma ions concentrations – Exp.1.

	Sodium	Chloride	Magnesium	Calcium
Ctrls	20	38	11	19
Cu10	16	50	−15	13
Cu100	30	56	21	−12

Differences (expressed as percentage) in plasma sodium, chloride, magnesium and calcium levels before and after the salinity transition in fish exposed to 0 (Ctrls), 10 and 100 µg/L copper.

#### NKA and CA2 gene expression

**Figure 3:**
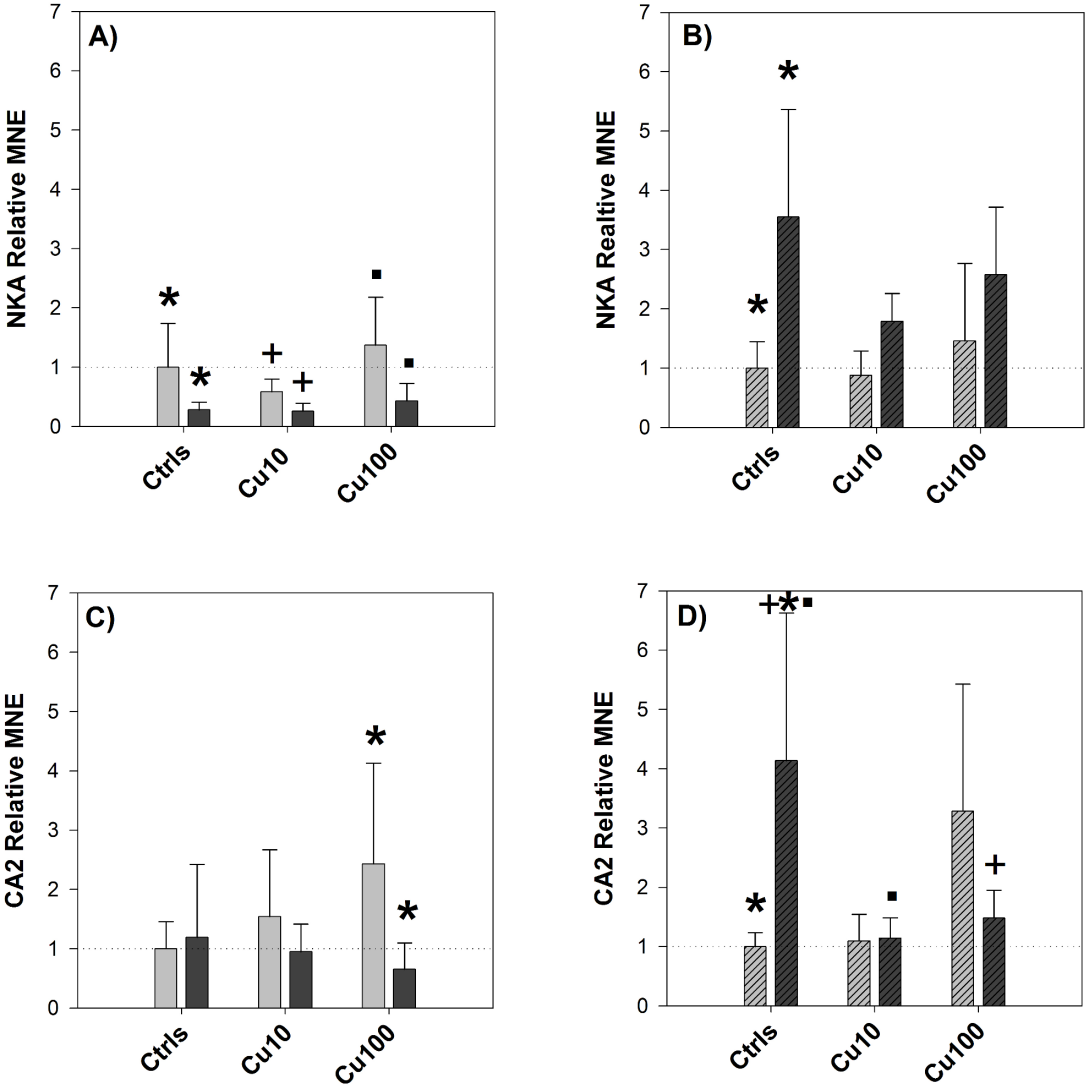
Pre-post change in NKA and CA2 expression – Exp.1. Relative Mean Normalized Expression (MNE) levels of NKA (graphs A and B), and CA2 (graphs C and D) measured in the gills (A and C) and in the mid-anterior tract of the intestine (B and D, striped bars) of sheepshead minnows exposed to 0 (Ctrls), 10 and 100 µg/L copper. Within each copper treatment, the pair of bars represents expression levels before (left side) and after (right side) the salinity transition. Relative MNE levels were determined by qPCR, normalized to control gene (18S) and expressed as fold change relative to pre-switch control value, which was set at 1 (dotted horizontal line). Values are means ± SE (*n* = 6/7). In each graph, bars sharing the same symbol (asterisk, cross or dot) are significantly different one from the other (*P*<0.05, Tukey *t*-test).

NKA gene expression did not appear to be significantly affected by copper exposure in a concentration-dependant fashion, either in the gills or in the intestine. However, when comparing NKA gene expression in controls before and after the salinity switch (Figure 3A and B), the change in salinity resulted in a significant down-regulation of NKA expression in the gills and a significant up-regulation (∼3.5-fold change) in the intestine. The same trend of down-regulation in the gills and up-regulation in the intestine was detected in the copper-exposed groups and in both tissues.

CA2 gene expression was affected by copper exposure in a concentration-dependant manner, both in gills and intestine, displaying in the high treatment a ∼2-fold up-regulation in the gills and a ∼3-fold up-regulation in the intestine. CA2 expression in the gills of the controls did not exhibit any substantial difference after the salinity switch, whereas in the intestine CA2 displayed a ∼4-fold up-regulation in the controls following the switch (Figure 3C and D).

### Experiment 2

#### Water chemistry

Concentrations of copper in the water were in the expected range for all treatments. Mean measured concentrations in the 32, 100 and 320 µg/L groups were, respectively, 31.82±9.1, 116.6±6.6 and 375.2±21 µg/L in the FW groups, and 29.01±4.7, 81.20±16 and 317.4±52 µg/L in the SW groups. Copper concentrations were <1 µg/L in all the control groups. Water chemistry parameters (Table S2) were stable over the exposure period. In the SW groups, mean measured salinity was 19.9±0.3 ppt, mean pH was only slightly higher than in FW and mean alkalinity was 180/240 mg/L CaCO_3_.

#### Plasma copper and sodium

**Figure 4:**
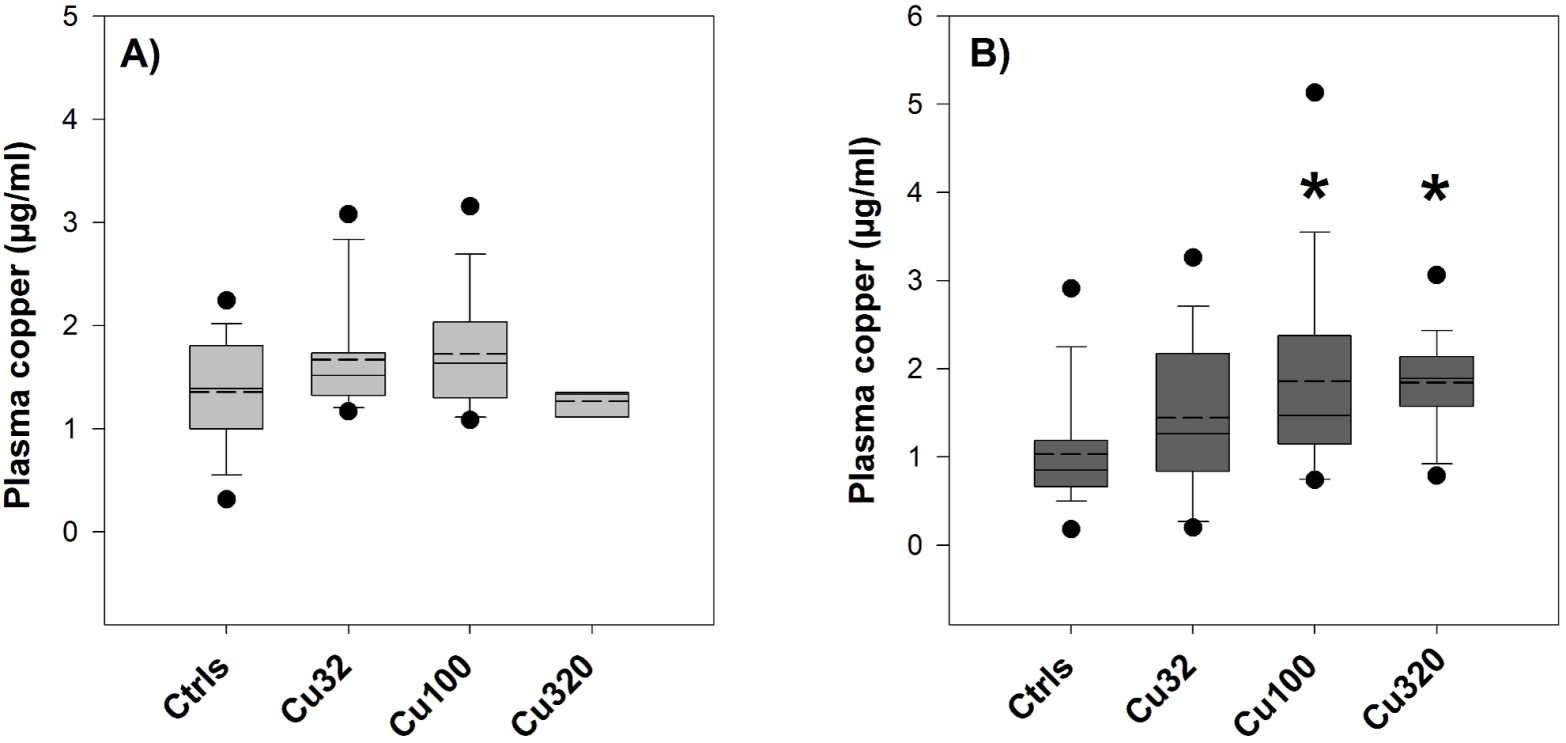
Plasma copper – Exp.2. Plasma copper levels (µg/ml) measured in fish exposed to 0 (Ctrls), 32, 100 and 320 µg/L copper in either FW (A) or SW (B). Values are means ± SE (*n* = 18/20, except for FW Cu320, where *n* = 3). The asterisk denotes statistically significant difference from the control value (*P*<0.05, Tukey t-test). Solid horizontal lines represent median values and dashed lines represent mean values.

The volume of plasma collected from the fish used in this experiment was lower than in the first experiment, due to the smaller fish size. Therefore, copper levels in plasma could be measured only where possible, given the dilution factor applied and the minimal sample volume required for the instrumental analysis. For this reason, results from fish sampled before and after the salinity switch within the same groups were pooled and reported graphically as one group (Figure 4), after verifying that there was no detectable difference between pre and post values. In the FW groups, plasma copper levels were slightly but not significantly elevated by copper exposure, whereas in the SW groups the mid- and high-copper treatments (Cu100 and Cu320) displayed significantly higher copper levels than controls.

**Figure 5:**
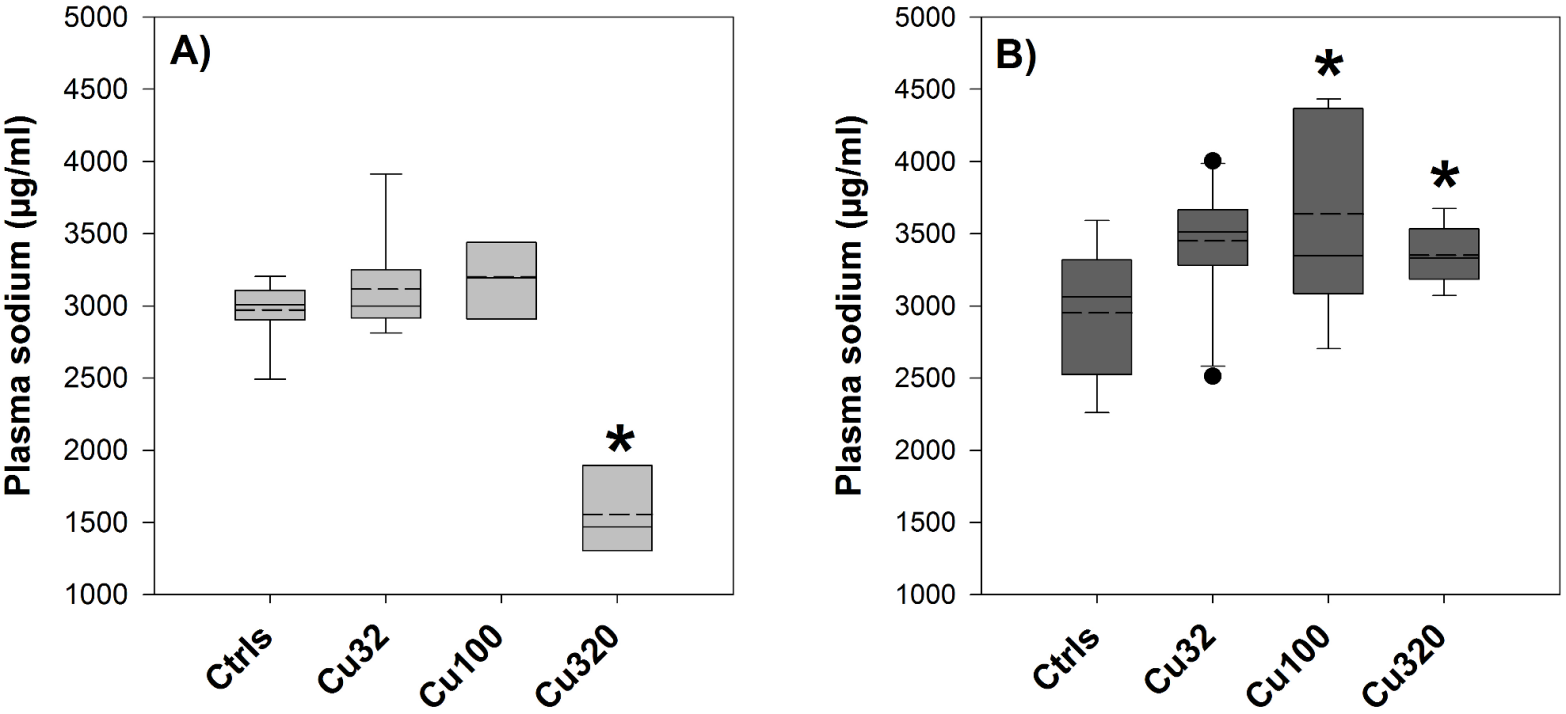
Plasma sodium – Exp.2. Plasma sodium concentrations (µg/ml) measured in fish exposed to 0 (Ctrls), 32, 100 and 320 µg/L copper in either FW (A) or SW (B). Values are means ± SE (*n* = 10, except for FW Cu320, where *n* = 3). The asterisk denotes statistically significant difference from the control value (*P*<0.05, Tukey *t*-test). Solid horizontal lines represent median values and dashed lines represent mean values.

Given the limited amount of plasma sample available in the second experiment, we chose to measure only sodium as the other plasma endpoint besides copper. Plasma sodium levels in FW and SW controls were very similar, despite the very different osmotic conditions, measuring respectively 2932±343.8 and 2952±459.2 µg/ml (equivalent to, respectively, 127.5±14.9 and 128.3±21.5 µmol/ml). Both in FW and SW conditions, the low- and mid-copper treatments (Cu32 and Cu100) displayed elevated sodium levels before the salinity transition, though this increase was statistically significant only in the SW Cu100 group (Figure 5). In the Cu320 treatments, a markedly different type of response was observed between the two salinity groups: in the SW one, sodium levels were significantly higher than controls, even though not higher than the Cu100 group, whereas in the FW group a 50% drop in plasma sodium levels was detected, although here *n* equalled only 3, because in the last days of exposure 6 fish were left in that group (out of 10), of which just 3 provided sufficient volume of plasma for the analysis. The FW Cu320 is the only group in the whole study were mortality was observed, presumably as a result of acute copper toxicity in freshwater conditions. However, despite the poor statistical power of the FW Cu320 group, the drastically lower levels measured in those samples were still significantly different from the control value. Unfortunately, in that treatment no fish survived the salinity transition, hence preventing any plasma analysis in that treatment following the switch.

**Figure 6:**
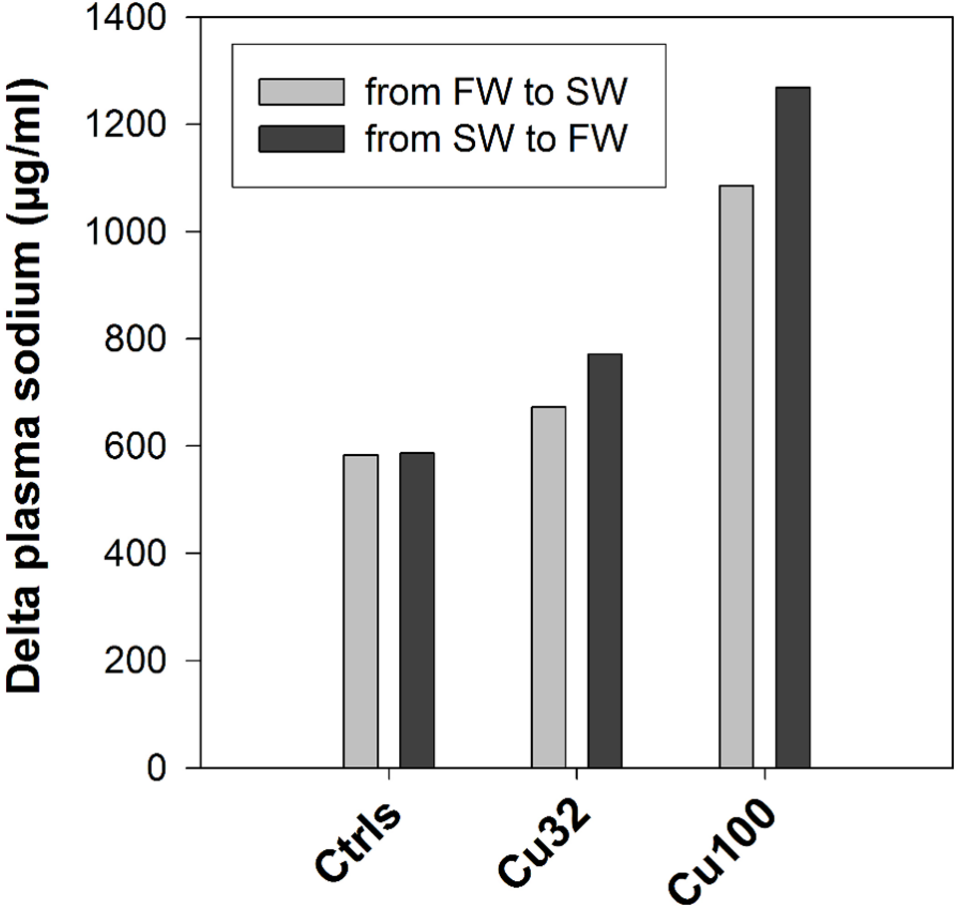
Degree of change in sodium levels. Difference (delta) in plasma sodium levels (µg/ml) before and after the salinity transition in fish exposed to 0 (Ctrls), 32 and 100 µg/L copper. Light grey and dark grey bars represent the delta change in the transition respectively from FW to SW and from SW to FW. No values are reported for the high-copper group as no fish survived after the salinity change. Values are calculated as difference between means and as such have no SE.

As for the pre-post switch effect on plasma sodium levels in the other treatments, a trend of response in ion homeostasis was observed. In the FW control group, mean sodium levels exhibited a 584 µg/ml increased after the salinity change, and in the SW control group almost exactly the same delta of plasma sodium (587 µg/ml) was detected before and after the salinity change (Figure 6), although in this case it represented a decrease. Hence, the degree of change in the controls was +20% for the FW groups and −20% for the SW ones. An almost equally symmetrical delta change was observed in the low-copper groups (Cu32), where the degree of change was around 22% (of either increase from FW to SW or decrease from SW to FW), and in the mid-copper groups, with a 34% increase in one direction and 32% decrease in the other.

#### NKA gene expression

**Figure 7:**
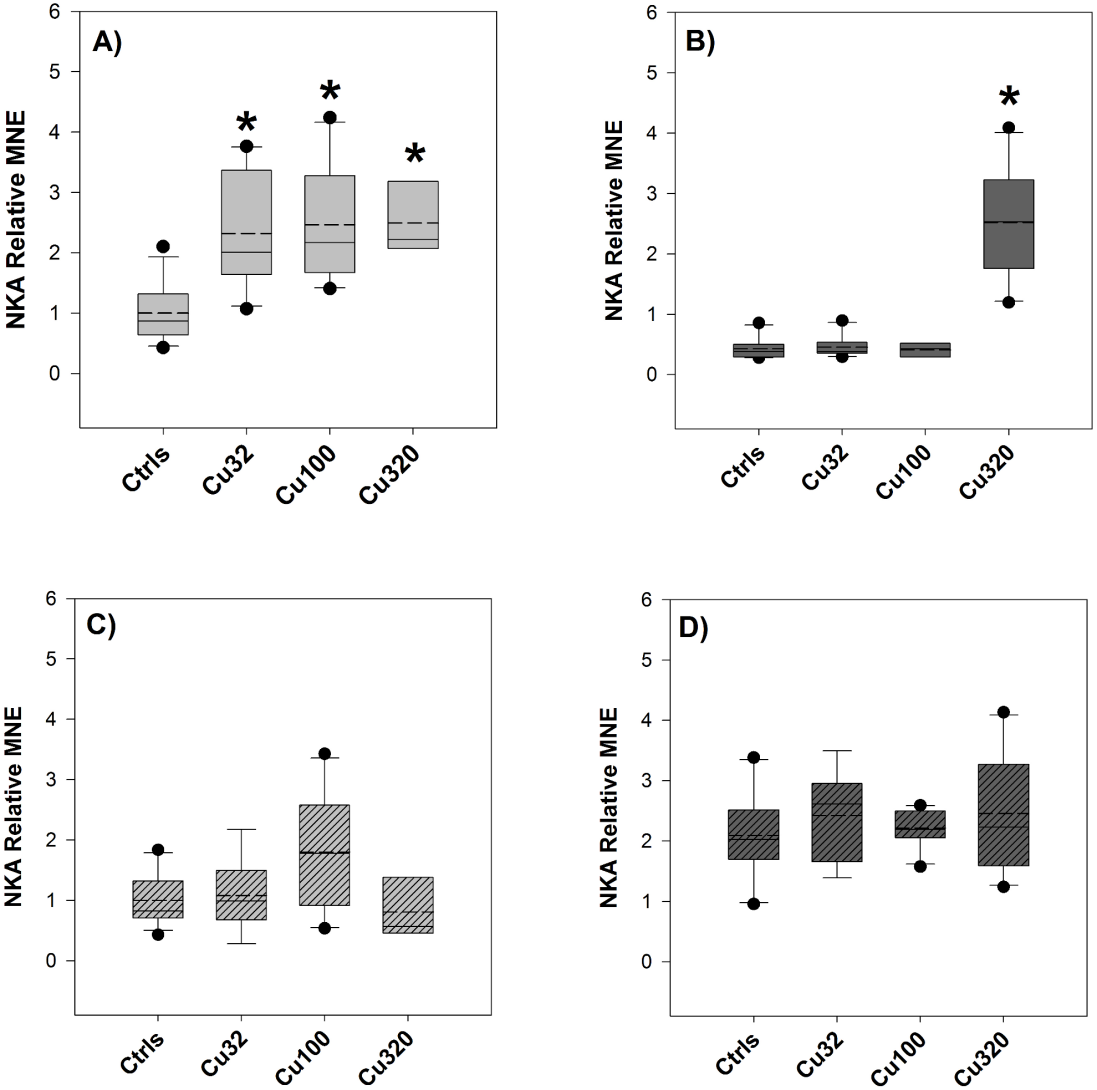
NKA expression – Exp.2. Relative Mean Normalized Expression (MNE) levels of NKA measured in the gills (A and B) and in the mid-anterior tract of the intestine (C and D, striped bars) of sheepshead minnows exposed to 0 (Ctrls), 32, 100 and 320 µg/L copper for 19 days. Light grey boxes (graphs A and C) and dark grey boxes (B and D) represent NKA levels respectively in FW and SW groups. Relative MNE levels were determined by qPCR, normalized to control gene (18S) and expressed as fold change relative to FW control value, which was set at 1. Values are means ± SE (*n* = 10/12, except for the FW Cu320 group, where *n* = 6). The asterisk denotes statistically significant difference from the control value (*P*<0.05, Tukey *t*-test). Solid horizontal lines represent median values and dashed lines represent mean values.

NKA gene expression was significantly up-regulated by copper exposure in the gills of both FW and SW groups, exhibiting a 2 to 3-fold change relative to control values (Figure 7A and B). In samples of intestine from FW groups, the low- and mid-copper groups displayed increased NKA expression, whilst in the high-copper group expression levels were down to control values (Figure 7C). No appreciable changes of NKA expression were detected in the intestine of SW groups (Figure 7D).

**Figure 8:**
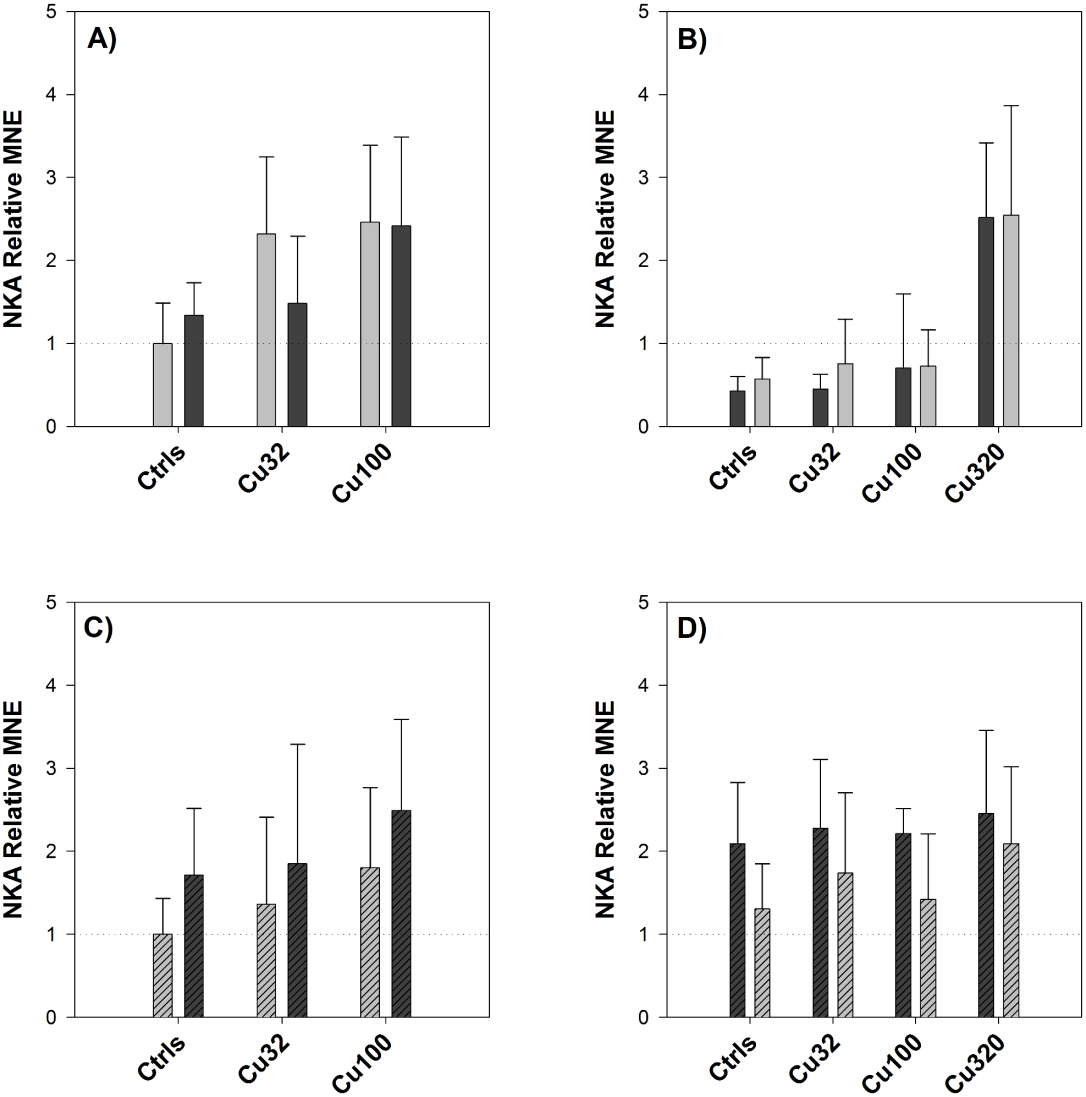
Pre-post change in NKA expression – Exp.2. Relative Mean Normalized Expression (MNE) levels of NKA measured in the gills (A and B) and in the mid-anterior tract of the intestine (C and D, striped bars) of sheepshead minnows exposed to 0 (Ctrls), 32, 100 and 320 µg/L copper. Within each copper treatment, the pair of bars represents NKA expression levels before (left side) and after (right side) the salinity transition. The colour of the bars represents the salinity conditions: light-grey for FW and dark-grey for SW. Relative MNE levels were determined by qPCR, normalized to control gene (18S) and expressed as fold change relative to pre-switch FW control value, which was set at 1 (dotted horizontal line). Values are means ± SE (*n* = 10/12). No values are reported for the high copper dose as no fish survived after the salinity change.

Considering the effects of the salinity change on NKA expression levels (Figure 8), a general trend of up-regulation from FW to SW and down-regulation from SW to FW was displayed in the intestine samples, whereas in the gills NKA expression did not change appreciably. However, when comparing NKA levels of expression in the gills of FW controls versus the SW controls, both of them before the salinity change, the FW gills exhibited a higher level of expression. The opposite was observed when comparing FW with SW controls in the intestine: in this tissue, the SW controls were the ones displaying higher expression levels.

#### CA2 gene expression

**Figure 9:**
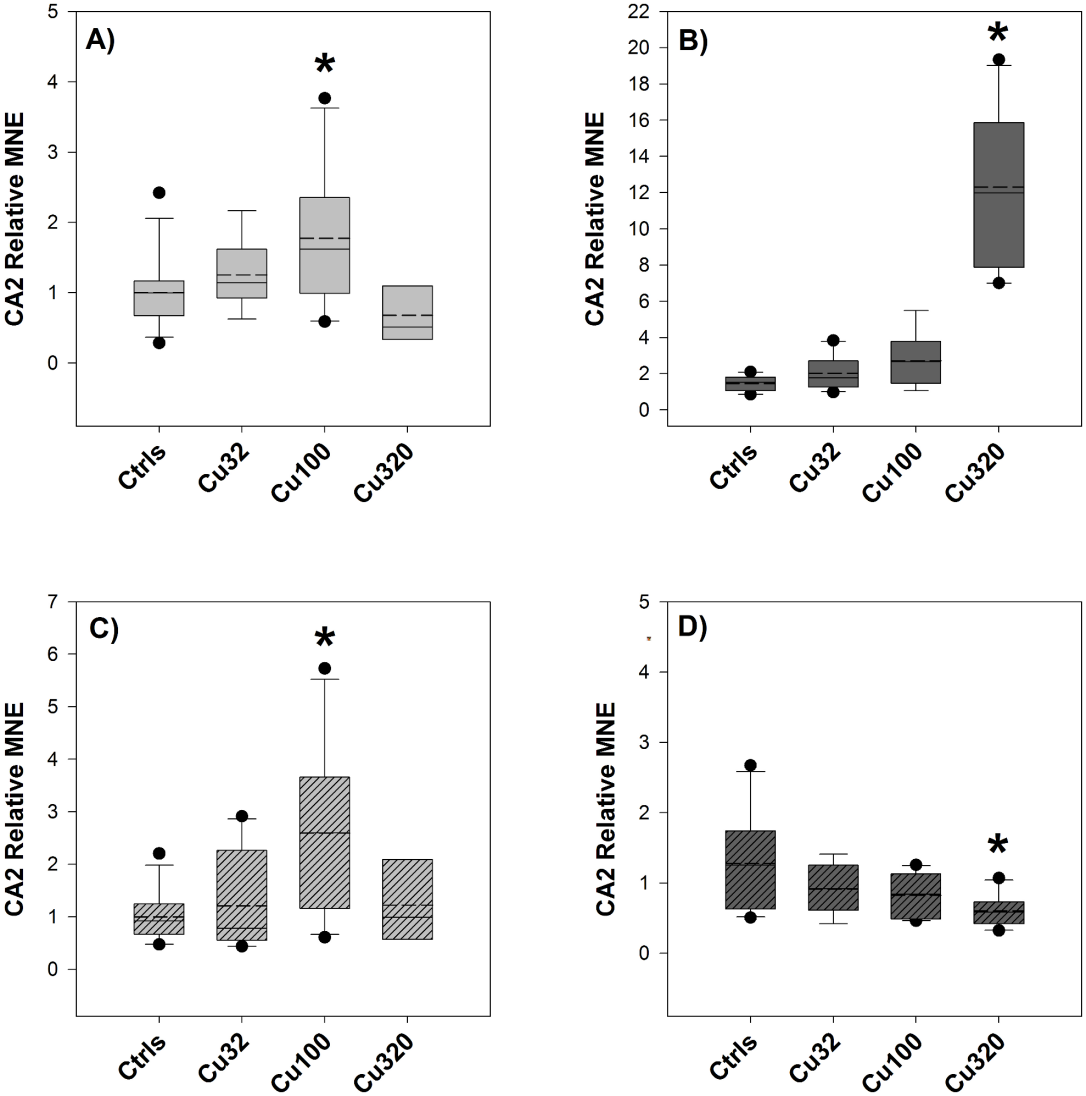
CA2 expression – Exp.2. Relative Mean Normalized Expression (MNE) levels of CA2 measured in the gills (A and B) and in the mid-anterior tract of the intestine (C and D, striped bars) of sheepshead minnows exposed to 0 (Ctrls), 32, 100 and 320 µg/L copper for 19 days. Light grey boxes (graphs A and C) and dark grey boxes (B and D) represent NKA levels respectively in FW and SW groups. Relative MNE levels were determined by qPCR, normalized to control gene (18S) and expressed as fold change relative to FW control value, which was set at 1. Values are means ± SE (*n* = 10/12, except for the FW Cu320 group, where *n* = 6). The asterisk denotes statistically significant difference from the control value (*P*<0.05, Tukey *t*-test). Solid horizontal lines represent median values and dashed lines represent mean values.

CA2 gene expression was up-regulated in response to copper exposure in the gills of both FW and SW groups, exhibiting a ∼2-fold change in the FW Cu100 group and a ∼12-fold change in the SW Cu320 group (Figure 9A and B), whereas in the FW Cu320 group CA2 levels dropped down to control values. Similarly, CA2 expression in intestine samples of FW groups was affected by copper in a concentration-dependant manner up to the mid-copper treatment, whilst the high-copper group broke the trend, displaying similar expression levels to the low-copper group (Figure 9C). CA2 expression in intestine samples of the SW groups displayed a trend of down-regulation in response to copper exposure, in contrast with the general up-regulation observed in the gills of both groups and in the intestine of the FW groups (Figure 9D).

**Figure 10:**
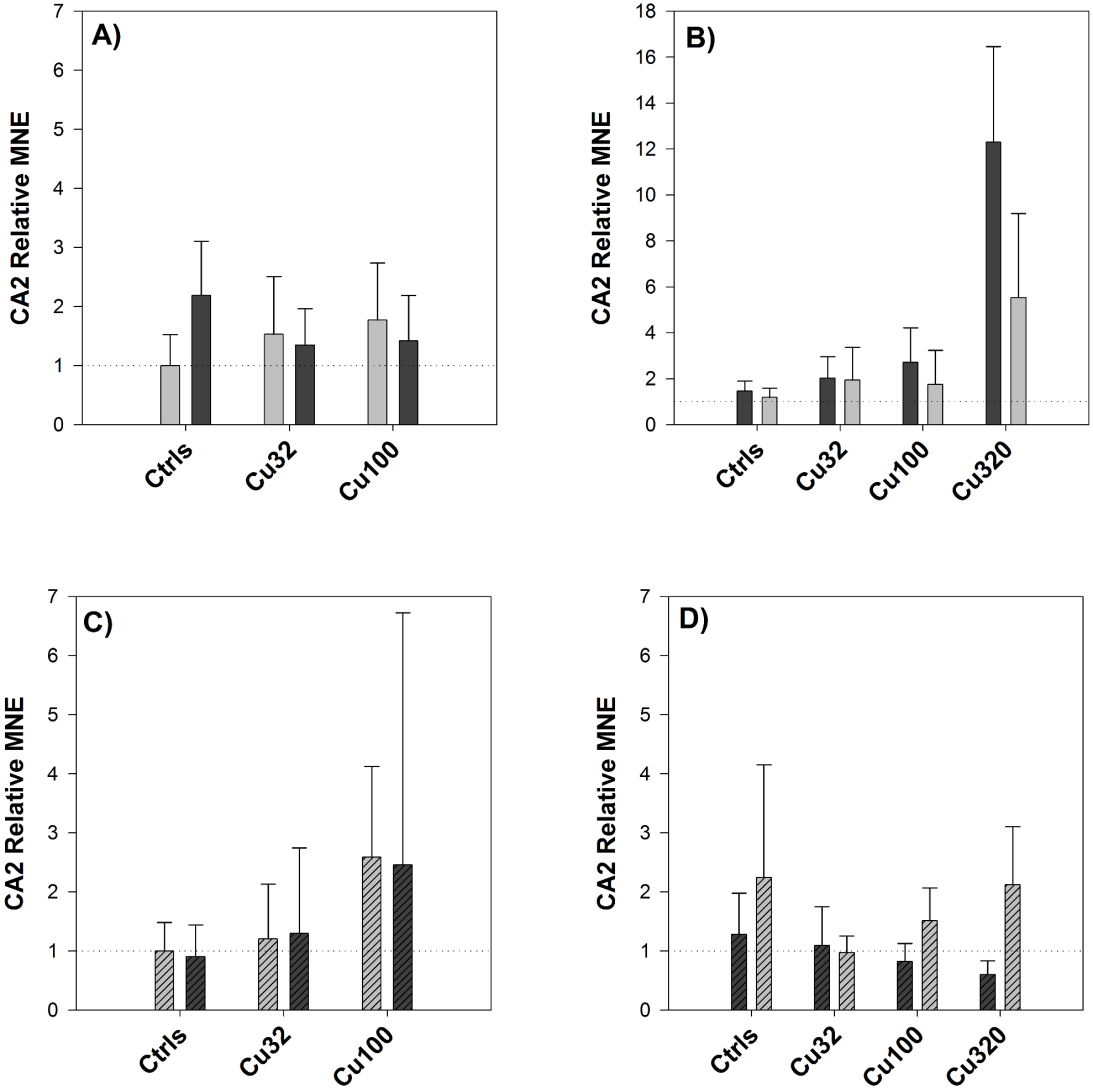
Pre-post change in CA2 expression – Exp.2. Relative Mean Normalized Expression (MNE) levels of CA2 measured in the gills (A and B) and in the mid-anterior tract of the intestine (C and D, striped bars) of sheepshead minnows exposed to 0 (Ctrls), 32, 100 and 320 µg/L copper. Within each copper treatment, the pair of bars represents CA2 expression levels before (left side) and after (right side) the salinity transition. The colour of the bars represents the salinity conditions: light-grey for FW and dark-grey for SW. Relative MNE levels were determined by qPCR, normalized to control gene (18S) and expressed as fold change relative to pre-switch FW control value, which was set at 1 (dotted horizontal line). Values are means ± SE (*n* = 10/12). No values are reported for the high copper dose as no fish survived after the salinity change.

No statistically significant change in CA2 expression was detected either in the gills or in the intestine in response to the salinity change, nor was it possible to observe any appreciable effect of copper on the response to the osmotic stress (Figure 10), in contrast to what was detected in the first experiment. However, a comparison between CA2 levels in controls of FW and SW revealed that the SW controls had a significantly higher expression levels, compared to the FW ones, whereas no appreciable difference was seen between FW and SW controls in the intestine.

## Discussion

Modelling of copper speciation in the exposure conditions revealed that a high proportion of the total dissolved copper was probably bound to organic matter and only a small percentage remained in the most bioavailable and hence toxic form of free metal ion (Table 2 and 3). Nevertheless, analyses of copper content in plasma showed that copper was still uptaken by fish in a concentration-related manner (Figure 1B and 4). One of the few studies that measured copper concentrations in the plasma reported only a small and transient increase of plasma copper levels after 24 hours of exposure, followed by a return to control values from day two onward [34]. This lack of a clear and consistent relationship between external concentrations and internal accumulation may appear to undermine the soundness of any biological effect attributed to water-borne metal exposure. However, metals are highly mobile chemical entities whose mode of action is intrinsically dynamic, in accordance with the high degree of complexation and compartmentalisation they undergo when entering the organism [35]. It is therefore their passing through some biological compartments, such as gills, rather than their accumulation, that bears most of their toxicological meaning and potential adverse effect, especially in short/mid-term exposures at low concentrations. They do accumulate in some tissues, mainly liver and bile [19], [36], but often in complexed and hence relatively inactive forms [37], whose toxicological relevance depends to the type of study and the endpoints of interest. This observation is supported by the high hepatic copper levels we detected in our study, possibly as a result of a copper-rich diet rather than of water-borne exposure (Figure 1A).

In the context of this study and its aims, the anchor point from where to start dissecting the results and build up their discussion is constituted by plasma ion homeostasis, as represented by plasma levels of sodium, chloride, calcium and magnesium, and their perturbation in response to copper and osmotic stress. All these four parameters responded to the salinity transition in the first experiment (Table 4); therefore, in the second experiment we chose to focus our attention only on sodium (Figure 5 and 6), which was used as a proxy of plasma ion homeostasis (or internal osmotic pressure). First of all, it is interesting to note that in both experiments and regardless of the exposure conditions (either FW or SW), control values of sodium in plasma before the salinity transition were all extremely close, around 3000 µg/ml, confirming that this parameter is very tightly and actively regulated by fish.

According to the literature, the iso-osmotic point of sheepshead minnow, meaning the salinity point at which the internal osmotic pressure equals that of the external medium, is around 10 ppt [38]. Based on this information, we should expect that both the fish held in FW and those held in SW (20 ppt) actively regulate their internal plasma concentrations against the same gradient of 10 ppt. However, we must also consider that our stocks of fish were bred and kept at 20 ppt for most of their life, before being adapted to FW conditions for a month. If we take into account this aspect, it is plausible to assume that strict FW conditions require a higher energetic cost to maintain the internal plasma ion homeostasis. Bearing this in mind, the first question we want to ask is whether the plasma sodium parameter was affected by copper exposure, in FW and in SW, irrespective of the salinity switch. Since copper is commonly considered an osmoregulatory disrupter [19], [39], displaying its osmoregulatory effects mainly through the disruption of sodium excretion mechanisms, we should expect that this impairment results in either increased sodium levels, when the gradient between internal and external plasma ion levels is negative (i.e. SW), or decreased sodium levels, when the gradient is positive (i.e. FW) [40]. And indeed, SW copper-exposed fish had higher levels of sodium in their plasma, compared to control ones, in so proving that copper exerted its osmoregulatory disruption, whereas in FW sodium levels displayed an increase in response to copper exposure, contrary to what we would expect given the positive osmotic gradient between internal and external osmotic pressure in these conditions. However, this small increase was observed only in the low- and mid-copper treatments, whereas in the high-copper treatment, Cu320, a dramatic drop of ∼50% in sodium levels was detected. It is important to stress here that in that particular group only 6 fish had survived after three weeks of copper exposure in FW: this in itself showed how those fish, exposed to the “double stress” of copper and FW conditions, were physiologically struggling, as confirmed by the 50% decrease in sodium levels. Given the tight regulation of this parameter under normal conditions, such a change can be considered as the effect of acute toxicity, similarly to other plasma parameters, such as human plasma glucose concentration [41]. This is an important element to consider when analysing this plasma sodium dataset with the aim of answering question *(1),* which is at the heart of the hypothesis we are testing in this study. To address this question we should look at the degree of change in plasma sodium levels before and after the salinity transition (Figure 6). If the degrees of change (or delta sodium) calculated for the controls are equal or similar to those calculated for the copper-exposed groups, then copper did not affect fish response to the osmotic stress. Alternatively, if the degrees of change, from FW to SW and from SW to FW, calculated for the copper-exposed groups are higher than those of the controls, we could conclude that copper did have an effect on the fish response to the osmotic stress and impaired their adaptation to the new conditions [20]–[21]. Results presented in Figure 6 not only support the latter option by showing a higher delta change in the copper treatments, but also display a concentration-dependent trend of increasing degrees of change at increasing copper concentrations, further supporting the hypothesis of a copper-disrupted osmoregulatory response to the salinity change. This was true in both directions of salinity transitions, from FW to SW and from SW to FW. One possible weakness of this set of results is the narrow range of change in plasma levels, i.e. few percentage points. However, this observation should be put in the context of this particular endpoint and its physiological regulation: as on the one side a 20% change was the “normal” degree of change observed in the controls, we know that on the other side a 50% change in plasma sodium is highly toxic for the organisms (see results in the Cu320 FW group). Framed in such context, we can consider a 22 or a 34% delta change as very physiologically significant.

Having addressed the question of whether or not copper affected the physiological response of fish to osmotic stress, the next step is to understand if the observed interaction is the combined effect of two independent effects, or if it is the outcome of a mechanistic interaction between the two stressors, i.e. copper and osmotic stress. According to the reasoning presented in the 2012 review [23], we hypothesize that the interaction between copper exposure and osmotic stress is indeed of a mechanistic nature. We also speculate that it is indirect, rather than direct, and that, in the exposure conditions applied in this study, it takes place mainly at the transcriptional level. In brief and according to our hypothesis, it should be an interaction rather than a combined effect because the pathways activated by both of them are similar (i.e. osmotic stress pathways) and they all share some common elements, one of which is the enzyme CA2. Given the numerous and diverse functional roles of CA2 [42]–[43], it is more plausible to think that copper affects the transcription of CA2 not directly, such as through the binding of some metal-responsive elements upstream of the gene coding for CA2, but rather indirectly, through the activation of osmotic-stress-related factors, which in turn regulate CA2 transcription. This indirect effect can be a form of compensatory response caused by impaired ionoregulation by copper exposure.

In order to interpret the results of the molecular analyses, it can be useful to apply the same set of questions used to interpret the results of plasma sodium levels, particularly *(a)* and *(c)*. The first question can be re-formulated as to whether copper exposure affected the transcriptional levels of CA2 and NKA, whereas the second one addresses the effect of copper on the transcriptional response to the osmotic stress. The expression of NKA was not significantly affected by copper exposure in the first experiment, in contrast with the second one, where there was a significant induction in response to copper in both FW and SW (Figure 7). CA2 expression was affected by copper in a dose-dependent manner, in both experiments and in both gills and intestine (Figure 9), demonstrating a maximum 12-fold up-regulation in the gills in response to copper (320 µg/L) in SW. As for the second question, i.e. whether copper affected the transcriptional response of fish to the salinity challenge, the two experiments provided similar results for NKA, whereas CA2 responses were somehow different between experiments. Considering the effect of copper on the regulation of NKA in response to the salinity transition, according to the results of both experiments (Figure 3 and 8) copper did not affect the response of NKA to the salinity change, as its regulation was not impaired by copper exposure. However, if we consider the results of the first experiment and compare the response of NKA in the two tissues, gills and intestine, it is interesting to note that the down-regulation of NKA caused by the salinity change in the gills was accompanied by an almost symmetrical up-regulation in the intestine, a pattern that might suggest a “deactivation” of NKA in one tissue and a parallel “activation” in the intestine as a result of the transition to SW conditions. This is in line with the expression patterns displayed by this isoform of NKA in rainbow trout during salinity transfer [43]. Considering the effect of copper on CA2 and its regulation in response to the salinity change, this is where some disagreements between the two datasets emerge. In the first experiment, CA2 expression in the intestine displayed a clear up-regulation in the controls following the salinity change (Figure 3C and D) and, notably, this up-regulation in the controls was not detected in the low-copper treatment and was even replaced by a down-regulation in the high-copper treatment. This opposite trend of response suggests that, under normal conditions, an osmotic stress induces the expression of CA2, at least in the intestine, but this response is disrupted by copper. Overall, these results support the original hypothesis that copper-exposed fish struggle to adapt to new salinity conditions because copper affects their osmoregulatory response to osmotic stress, and it does so by interfering with the regulation of osmotic effector proteins such as CA2. However, contrary to what was observed in the first experiment, the results from the second experiment did not show any significant effect of copper on the regulation of CA2 either in the gills or in the intestine (Figure 10).

We speculate that the disagreement between the two datasets can be explained by addressing the interaction of copper and salinity from a multi-stressor perspective, where copper exposure and osmotic stress are regarded as a chemical and an environmental stressor that disrupt the homeostasis of the system, i.e. the fish. In this systemic context, it is plausible to assume that the organism responds to the perturbation of its homeostasis by activating responses that are of a magnitude and complexity appropriate to the degree of perturbation, i.e. level of stress. Put another way, if the organism responded to a mild stress with a disproportionally complex response, it would waste its cellular resources, whereas underestimating the level of stress may compromise its cellular function. Therefore, it is energetically sensible for the organism to size its adaptive response to the severity of the perturbation. Applying this concept to the case of osmotic stress responses, we should expect that different doses of osmotic stress elicit different magnitudes of response, as was indeed shown by our results on CA2 transcriptional levels in the two experiments. Since osmoregulatory mechanisms are finely modulated according to varying degrees of osmotic stress [44], the different rate at which the salinity transition was performed in the two exposures (4 hours in the first and 8 hours in the second one) could explain the activation of different transcriptional responses, resulting in a significant induction of CA2 in the first experiment and in an overall unaffected response of the same enzyme in the second one. Additionally, the responses of some endpoints at high copper doses were completely different than those displayed at low and mid doses, suggesting that somewhere between the mid and the high dose a threshold of different pathways activation was passed, as the dose of chemical stress applied went from mild to severe. Of course we are aware that this is another way to formulate the classical concept of chronic and acute dose, but this may actually need to be reformulated, when multiple stressors of different source and nature are studied in atypical combinations. Although these arguments are admittedly speculative, they in any case hint at the complexity of multi-stressor studies. Since such a complexity lies at the level of biological responses to different stressors, it cannot be overlooked when it comes to modelling metal toxicity in multi-stressor scenarios.

## Supporting Information

Figure S1 **Salinity switch – Exp.2.** Salinity measurements (ppt) throughout the salinity transition period (8 h) of Experiment 2. The graph on the left side represents the FW groups in the 4 treatments (Controls, Cu32, Cu100 and Cu320) that were moved towards SW conditions, whilst the graph on the right represents the SW groups moved towards FW. Control groups (white circles) were started at 10 am, Cu32 (grey triangles) at 12 am, Cu100 (dark grey squares) at 14 pm and Cu320 (black diamonds) at 16 pm. A 2 h-lag was chosen to ensure that all treatments were held in the new conditions for exactly 24 h prior to sampling on the following day.
(TIF)

Figure S2 **Exposure set-ups – Exp.2.** Exposure set-ups one on one side of the exposure room (the other was symmetrical), respectively before (top figure) and after (bottom figure) the salinity switch. Blue colour represents the saltwater (SW) supply and dark green colour the dilution water (DW) supply. The SW stock was mixed with a RZR 2052 overhead stirrer (Heidolph) in two 80 L tanks (one per side) and then transferred into two 40 L tanks, from which the SW was dosed at a rate of 12 ml/min via a peristaltic pump into the mixing chambers of the SW groups. Copper stock solutions were dosed at a rate of 0.06 ml/min via one single peristaltic pump that fed both sides of the room.
(TIF)

Table S1 **Water parameters in fish tanks – Exp.1.** Reported values are means ± SD of the measurements taken daily in all 6 groups (*n* = 6) respectively before (PRE) and after (POST) the salinity switch.
(DOCX)

Table S2 **Water parameters in fish tanks – Exp.2.** Reported values are means ± SD of the measurements taken daily over the exposure period, respectively in the freshwater (FW) groups and in the saltwater (SW) groups.
(DOCX)

Table S3 **TOC and major cations concentrations in the water – Exp.1.** Experiment 1 water concentrations (µg/mL) of Total Organic Carbon (TOC) and Na^+^, Ca^2+^ and Mg^2+^ analysed respectively by Shimadzu total organic carbon-V CPN Analyzer and F-AAS. Reported FW and SW values are means ± SD of all 6 groups (*n* = 6) respectively before (PRE) and after (POST) the salinity switch.
(DOCX)

Table S4 **Copper concentrations in the water – Exp.1.** Experiment 1 water concentrations (µg/L) of copper during the exposure period, analysed by GF-AAS (controls and 10 µg/L) and F-AAS (100 µg/L). Reported values are means ± SD (*n* = 3 for each given time point and concentration).
(DOCX)
