# Supplementary figures and images for: A New Role for Carbonic Anhydrase 2 in the Response of Fish to Copper and Osmotic Stress: Implications for Multi-Stressor Studies
Source: PLoS One. 2014 Oct 1;9(10):e107707. doi: 10.1371/journal.pone.0107707 (PMC4182668; doi:10.1371/journal.pone.0107707)

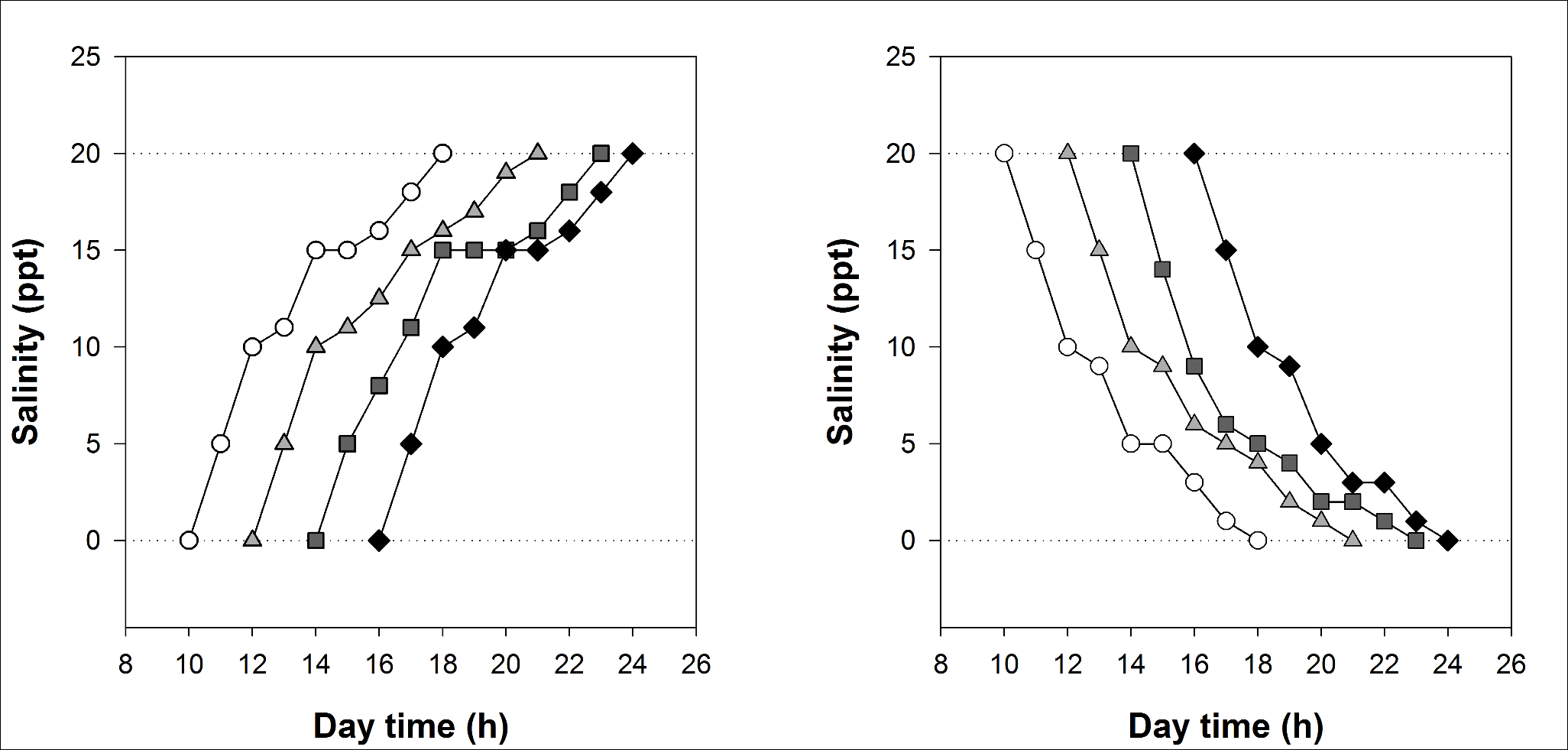

Supplement: Figure S1 — Salinity switch – Exp.2. Salinity measurements (ppt) throughout the salinity transition period (8 h) of Experiment 2. The graph on the left side represents the FW groups in the 4 treatments (Controls, Cu32, Cu100 and Cu320) that were moved towards SW conditions, whilst the graph on the right represents the SW groups moved towards FW. Control groups (white circles) were started at 10 am, Cu32 (grey triangles) at 12 am, Cu100 (dark grey squares) at 14 pm and Cu320 (black diamonds) at 16 pm. A 2 h-lag was chosen to ensure that all treatments were held in the new conditions for exactly 24 h prior to sampling on the following day. (TIF) [file pone.0107707.s001.tif]

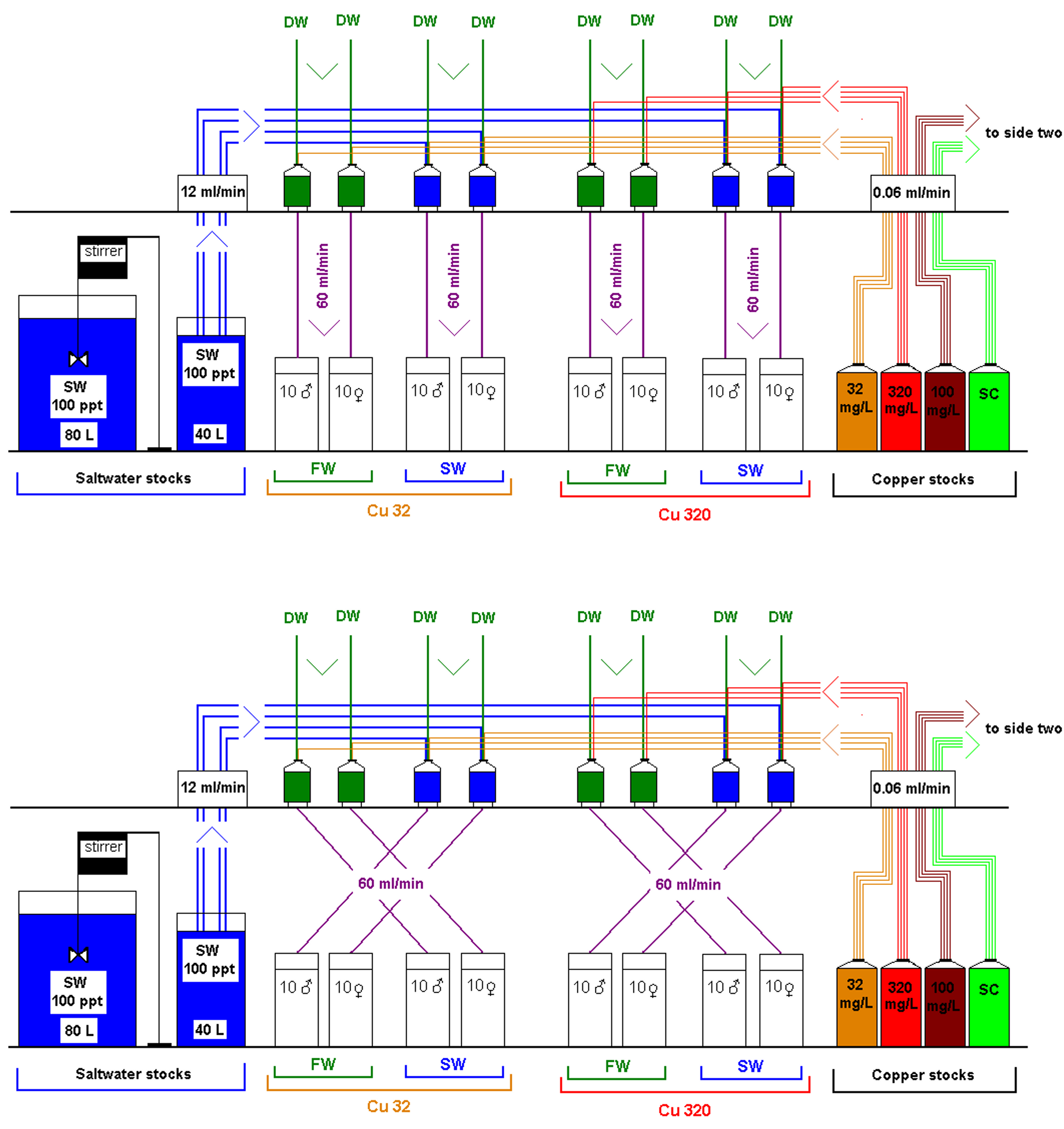

Supplement: Figure S2 — Exposure set-ups – Exp.2. Exposure set-ups one on one side of the exposure room (the other was symmetrical), respectively before (top figure) and after (bottom figure) the salinity switch. Blue colour represents the saltwater (SW) supply and dark green colour the dilution water (DW) supply. The SW stock was mixed with a RZR 2052 overhead stirrer (Heidolph) in two 80 L tanks (one per side) and then transferred into two 40 L tanks, from which the SW was dosed at a rate of 12 ml/min via a peristaltic pump into the mixing chambers of the SW groups. Copper stock solutions were dosed at a rate of 0.06 ml/min via one single peristaltic pump that fed both sides of the room. (TIF) [file pone.0107707.s002.tif]
