# Supplementary material for: A New Role for Carbonic Anhydrase 2 in the Response of Fish to Copper and Osmotic Stress: Implications for Multi-Stressor Studies
Source: PLoS One. 2014 Oct 1;9(10):e107707. doi: 10.1371/journal.pone.0107707 (PMC4182668; doi:10.1371/journal.pone.0107707)
Supplement: Table S1 — Water parameters in fish tanks – Exp.1. Reported values are means ± SD of the measurements taken daily in all 6 groups (n = 6) respectively before (PRE) and after (POST) the salinity switch. (DOCX) [file pone.0107707.s003.docx]

**Table S1.** **Water parameters in fish tanks – Exp.1**. Reported values are means ± SD of the measurements taken daily in all 6 groups (*n* = 6) respectively before (PRE) and after (POST) the salinity switch

|  | PRE | POST |
| --- | --- | --- |
| Salinity (ppt) | 0 | 20 |
| Temperature (°C) | 25.8 ± 0.23 | 25.3 ± 0.25 |
| pH | 7.9 ± 0.08 | 8.4 ± 0.06 |
| Oxygen (mg/L) | 7.2 ± 0.34 | 7.9 ± 0.06 |
| Alkalinity (mg/L CaCO_3_) | 220 | 260 |
