# Supplementary material for: A New Role for Carbonic Anhydrase 2 in the Response of Fish to Copper and Osmotic Stress: Implications for Multi-Stressor Studies
Source: PLoS One. 2014 Oct 1;9(10):e107707. doi: 10.1371/journal.pone.0107707 (PMC4182668; doi:10.1371/journal.pone.0107707)
Supplement: Table S2 — Water parameters in fish tanks – Exp.2. Reported values are means ± SD of the measurements taken daily over the exposure period, respectively in the freshwater (FW) groups and in the saltwater (SW) groups. (DOCX) [file pone.0107707.s004.docx]

**Table S2. Water parameters in fish tanks – Exp.2.** Water parameters measured in fish tanks during Experiment 2. Reported values are means ± SD of the measurements taken daily over the exposure period, respectively in the freshwater (FW) groups and in the saltwater (SW) groups.

|  | FW | SW |
| --- | --- | --- |
| Salinity (ppt) | 0 | 19.9 ± 0.3 |
| Temperature (°C) | 24.8 ± 0.4 | 24.4 ± 0.5 |
| pH | 7.57 ± 0.01 | 7.59 ± 0.06 |
| Oxygen (mg/L) | 6.00 ± 2.3 | 5.76 ± 2.2 |
| Alkalinity (mg/L CaCO_3_) | 120/180 | 180/240 |
