# Supplementary material for: A New Role for Carbonic Anhydrase 2 in the Response of Fish to Copper and Osmotic Stress: Implications for Multi-Stressor Studies
Source: PLoS One. 2014 Oct 1;9(10):e107707. doi: 10.1371/journal.pone.0107707 (PMC4182668; doi:10.1371/journal.pone.0107707)
Supplement: Table S3 — TOC and major cations concentrations in the water – Exp.1. Experiment 1 water concentrations (µg/mL) of Total Organic Carbon (TOC) and Na+, Ca2+ and Mg2+ analysed respectively by Shimadzu total organic carbon-V CPN Analyzer and F-AAS. Reported FW and SW values are means ± SD of all 6 groups (n = 6) respectively before (PRE) and after (POST) the salinity switch. (DOCX) [file pone.0107707.s005.docx]

**Table S3.** **TOC and major cations concentrations in the water – Exp.1.** Experiment 1 water concentrations (µg/mL) of Total Organic Carbon (TOC) and Na^+^, Ca^2+^ and Mg^2+^ analysed respectively by Shimadzu total organic carbon-V CPN Analyzer and F-AAS. Reported FW and SW values are means ± SD of all 6 groups (*n* = 6) respectively before (PRE) and after (POST) the salinity switch.

|  | PRE | POST |
| --- | --- | --- |
| TOC | 2.61 ± 0.55 | |
| Na^+^ | 32.50 ± 0.42 | 6367 ± 211 |
| Mg^2+^ | 4.580 ± 0.04 | 497.9 ± 50.6 |
| Ca^2+^ | 101.7 ± 1.63 | 258.0 ± 18.6 |
