# Supplementary material for: A New Role for Carbonic Anhydrase 2 in the Response of Fish to Copper and Osmotic Stress: Implications for Multi-Stressor Studies
Source: PLoS One. 2014 Oct 1;9(10):e107707. doi: 10.1371/journal.pone.0107707 (PMC4182668; doi:10.1371/journal.pone.0107707)
Supplement: Table S4 — Copper concentrations in the water – Exp.1. Experiment 1 water concentrations (µg/L) of copper during the exposure period, analysed by GF-AAS (controls and 10 µg/L) and F-AAS (100 µg/L). Reported values are means ± SD (n = 3 for each given time point and concentration). (DOCX) [file pone.0107707.s006.docx]

**Table S4. Copper concentrations in the water – Exp.1.** Experiment 1 water concentrations (µg/L) of copper during the exposure period, analysed by GF-AAS (controls and 10µg/L) and F-AAS (100µg/L). Reported values are means ± SD (*n* = 3 for each given time point and concentration).

| Exposure day | Replicate tank | Nominal copper concentrations (µg/L) | | |
| --- | --- | --- | --- | --- |
|  |  | Control | 10 | 100 |
| 3 | A | <1µg/L | 10.7 ± 5.7 | 103.5 ± 3.70 |
|  | B | <1µg/L | 8.74 ± 3.5 | 115.7 ± 0.80 |
| 6 | A | <1µg/L | 11.2 ± 3.7 | 110.6 ± 1.96 |
|  | B | <1µg/L | 9.34 ± 2.1 | 123.2 ± 1.19 |
| 8 | A | <1µg/L | 9.70 ± 0.2 | 120.4 ± 7.33 |
|  | B | <1µg/L | 10.5 ± 0.7 | 125.8 ± 2.83 |
| 9 | A | <1µg/L | 10.5 ± 5.7 | 98.09 ± 0.19 |
|  | B | <1µg/L | 9.63 ± 2.3 | 127.1 ± 0.23 |
| mean values (µg/L) | | | 10.04 ± 0.77 | 115.5 ± 9.97 |
